# *Lactobacillus paracasei* Jlus66 extenuate oxidative stress and inflammation via regulation of intestinal flora in rats with non alcoholic fatty liver disease

**DOI:** 10.1002/fsn3.1118

**Published:** 2019-07-15

**Authors:** Wei Wang, Qian Li, Wenhui Chai, Chunyan Sun, Tiehua Zhang, Changhui Zhao, Yuan Yuan, Xinyu Wang, Huiqin Liu, Haiqing Ye

**Affiliations:** ^1^ College of Food Science and Engineering, Jilin University Changchun China; ^2^ Jilin Provincial People's Hospital Changchun China

**Keywords:** inflammation, intestinal flora, nonalcoholic fatty liver disease, oxidative stress

## Abstract

The nonalcoholic fatty liver disease (NAFLD) is a progressive liver disease that affects the health of people in an increasing rate. In the current research, we investigated the beneficial effect of a novel probiotic strain *L. paracasei* Jlus66 (Jlus66) on rats with high‐fat diet (HFD)‐induced NAFLD. The intestinal flora of rats was analyzed based on V3‐V4 region 16S rDNA sequencing. Moreover, we measured the oxidative stress and inflammation factors in the liver using commercial ELISA kit, and the lipopolysaccharide (LPS) in serum with chromogenic end‐point tachypheus amebocyte lysate. Compared with the HFD‐induced group, Jlus66 treatment significantly decreased the malondialdehyde (MDA) level in the serum (*p* < 0.05). Additionally, Jlus66 significantly enhanced the activities of superoxide dismutase (SOD) and glutathione peroxidase (GSH‐Px) in the liver and serum (*p* < 0.05). Jlus66 administration also reduced the levels of tumor necrosis factor (TNF‐α) and interleukin‐6 (IL‐6), and inversely increased the interleukin‐10 (IL‐10) level in serum (*p* < 0.05). Intestinal flora analysis results showed that Jlus66 can improve intestinal flora structure by increasing the abundance of gram‐positive flora such as Firmicutes, and decreasing gram‐negative flora such as Bacteroidetes, Proteobacteria, and Fusobacteria, and then reduced LPS concentration in the serum. So we concluded that Jlus66 can improve NAFLD by modulating the intestinal flora and followed reduction of oxidative stress (OxS) and inflammation.

## 1 INTRODUCTION

NAFLD is a complicated progressive liver disease that is caused by multiple factors (Bellentani, Scaglioni, Marino, & Bedogni, 2010) and is generally considered by the scientific community as hepatic expression of the metabolic syndrome along with chronic systemic OxS. Fat accumulation in the liver augments its vulnerability to OxS, followed by inflammation. OxS occurs via elevated formation of reactive oxygen species (ROS), which initiates lipid peroxidation by targeting the double bonds of polyunsaturated fatty acid. The subsequent formation of extremely reactive aldehyde components, namely 4‐hydroxy‐2‐nonenal and MDA, causes intracellular damage (Spahis, Delvin, Borys, & Levy, 2017). Concomitantly, antioxidant components such as catalase, glutathione (GSH), GSH S‐transferase, SOD, and coenzyme Q begin to decline (Erhardt et al., 2011; Videla et al., 2004). Hepatic inflammatory stress is a critical event in the “second hit” stage of NAFLD. Interleukin‐1β (IL‐1β), IL‐6, and TNF‐α are crucial proinflammatory cytokines produced by injured hepatocytes, immune cells, and activated Kupffer cells, which play a vital role in inflammation. IL‐1β can induce hepatocyte steatosis. TNF‐α and IL‐6 levels correlate with the severity of inflammation, fibrosis, and histological changes in the liver (Chen et al., 2019). Therefore, the effective approach to treat NAFLD is to target the oxidative stress, inflammation, and injury that stress the NAFLD subjects (Rotman & Sanyal, 2017). Though the mechanism of NAFLD is not fully elucidated, it is believed that oxidative stress plays a key role in the development of hepatocyte injury associated with NAFLD (El Hadi, Vettor, & Rossato, 2018; Masarone et al., 2018), followed by branches of several intracellular events as well as extracellular factors such as dysbiosis of the gut flora (Spahis et al., 2017).

Gut microbiota dysbiosis can result in the development of obesity‐related NAFLD. Although it is unclear whether the microbiota have a major impact on the incidence of NAFLD, the relative abundance of certain bacterial groups (Marchesi et al., 2016), the presence of harmful micro‐organisms, the metabolic function of microbes, host genetics, and/or combinations of these factors are important in the pathogenesis of NAFLD. Consistently, patients with NAFLD have slight intestinal bacterial overgrowth and increased intestinal permeability (Miele et al., 2009). Changes in the levels of plasma metabolites that relate to immunological responses occur during probiotic treatment (Martin et al., 2006). Immune modulation by probiotics is presumed to be one of the main mechanisms of probiotic action in human health (Guarner & Malagelada, 2003). The increase in beneficial bacteria and restoration of gut barrier function can promote the health status of the host and subsequently decrease the risk of diseases (vanWinsen et al., 2002). The consensus theory is that the disturbed interaction between the intestinal epithelium and some symbiotic bacteria induces rapid production of ROS, which may lead to the pathogenesis of NAFLD (Borrelli et al., 2018). In addition, fecal microbiota transplantation has been approved for the treatment of *Clostridium difficile* infection, metabolic syndrome, and irritable bowel syndrome (Woodhouse, Patel, Singanayagam, & Shawcross, 2018).

Jlus66 was recently isolated from the local “milk pimple” originated in Jilin Province in northeast China. Jlus66 has been shown to have probiotic properties and lipid‐lowering ability in the previous study (Ye et al., 2017). In this study, to further investigate the beneficial effects of Jlus66 on NAFLD, the intestinal flora, oxidative stress, LPS, and inflammatory factors in the serum/liver were evaluated in rats with HFD‐induced NAFLD.

## 2 MATERIALS AND METHODS

### 2.1 Materials and reagents

De Man Rogosa Sharp (MRS) broth (HB0384‐1) was purchased from Qingdao Hope Bio‐technology Co., Ltd; SOD (A001‐1–2, Hydroxylamine method), MDA (A003‐1–2, TBA method), GSH‐Px (A005‐1–2, Colorimetric method) assay kits and TNF‐α (H052), IL‐10 (H009), and IL‐6 (H007) ELISA kits were purchased from Nanjing Jiancheng Bioengineering Institute (Nanjing, China); LPS assay kit (fs‐E1848, chromogenic end‐point tachypheus amebocyte lysate) was purchased from Xiamen Bio‐Endo Technology Co., Ltd.

### 2.2 Strain preparation and animal experiments

Jlus66 was amplified with 10% (v/v) MRS at 37°C for 24 hr; the bacteria were collected by centrifuging (4,000 g, 20 min) and diluted to 1, 2, 4×10^10 ^cfu. All biological samples used in this study were from the same batch of experimental animals as previous report (Ye et al., 2017). Animal experiment was performed in compliance with the National Institute of Health Guidelines for the Care and Use of Laboratory Animals (National Research Council, 1996) and was approved by Institutional Animal Care and Use Committee of Jilin University (IACUC). Briefly, 40 specific pathogen‐free male Wistar rats (200 ± 10 g, Yisi Experimental Animal Technology Co., Ltd) were maintained in an air‐conditioned room at 20–24°C, with 20%–40% relative humidity. The rats had free access to food and water, and kept in 12‐h light–dark cycle. After two weeks of new environment acclimation, rats were divided into five groups randomly (2 rats per cage), named Con, HFD, ProL, ProM, and ProH groups, respectively. The Con group was fed with standard diet, while the HFD and the JLus66 treatment groups were given HFD (Product #SPF; Yisi, Changchun, China). The HFD contains 10% moisture, 243 g/kg protein, 297 g/kg carbohydrate, and 360 g/kg fat. In addition, carbohydrate was provided from wheat flour, protein sources included casein, 0.3% DL‐methionine, and 0.5% L‐cysteine, vitamin and mineral concentrations met the requirements for standard rat diets, all essential amino acids were present at levels that met or exceeded National Research Council recommended, as previously described (Esposito et al., 2009). In addition, the JLus66 treatment groups were orally administered 1, 2, 4 × 10^10 ^cfu of Jlus66 daily, while the other two groups (HFD and Con) were orally administered equal amount of distilled water.

After 20 weeks of administration, the rats were sacrificed under barbital sodium anesthesia. The blood was collected from abdominal femoral artery use 5‐ml centrifuge tubes; serum was separated by centrifuging at 1,250 *g*, 4℃ for 10 min. The livers were cut to fit into the cryotube. The cecal content was collected for intestinal flora analysis. Specifically, six rats were selected from eight of each group randomly, two rats cecal contents of the same group were pooled as one sample, and three final samples were formed in each group. All samples were stored at −80°C for sequent analysis. Each experiment has been repeated at minimum three times.

### 2.3 Biochemical analysis

One gram liver of each rat was homogenized on ice with 9 ml Tris–HCl (pH: 7.4) and then centrifuged at 1,000 *g* for 15 min at 4°C to collect supernatant. The levels of MDA, SOD, GSH‐Px, TNF‐α, IL‐10, IL‐6, and LPS in the serum or supernatants were determined using commercial kits according to the instructions.

DNA from cecal contents was extracted using the Micro‐Elute Genomic DNA Kit (D3096‐01, Omega, Inc.), eluted in 50 µl Elution buffer, and sequenced by LC‐Bio Technology Co., Ltd. The V3‐V4 region of the prokaryotic (bacterial and archaeal) small‐subunit (16S) rRNA gene was amplified with slightly modified versions of primers 338F (5'‐ACTCCTACGGGAGGCAGCAG‐3') and 806R (5'‐GGACTACHVGGGTWTCTAAT‐3') (Fadrosh et al., 2014). The 5' ends of the primers were tagged with specific barcodes per sample and sequencing universal primers. PCR amplification was performed in a total volume of 25 µl reaction mixture containing 25 ng of template DNA, 12.5 µl PCR Premix, 2.5 µl of each primer, and PCR‐grade water to adjust the volume. The PCR conditions to amplify the prokaryotic 16S fragments consisted of an initial denaturation at 98°C for 30 s; 35 cycles of denaturation at 98℃ for 10 s, annealing at 54℃/52℃ for 30 s, and extension at 72℃ for 45 s; and then final extension at 72℃ for 10 min. The PCR products were identified by 2% agarose gel electrophoresis and standardized through AxyPrep^TM^ Mag PCR Normalizer (Axygen Biosciences). The amplicon libraries were prepared with AM Pure XT beads (Beckman Coulter Genomics), and the size and quantity were evaluated with LabChip GX (Perkin Elmer) and Illumina's Library Quantitative Kit (Kapa Biosciences). The libraries were sequenced on 300 PE MiSeq runs, and one library was sequenced with both protocols using the standard Illumina sequencing primers.

Samples were sequenced on an Illumina MiSeq platform according to the manufacturer's recommendations, provided by LC‐Bio. Paired‐end reads were assigned to samples based on their unique barcode and truncated by cutting off the barcode and primer sequence. Paired‐end reads were merged using FLASH. Quality filtering on the raw tags was performed under specific filtering conditions to obtain the high‐quality clean tags according to the FastQC (V 0.10.1). Chimeric sequences were filtered using Verseach software (v2.3.4). Sequences with ≥97% similarity were assigned to the same operational taxonomic units (OTUs) by Verseach (v2.3.4). Representative sequences were chosen for each OTU, and taxonomic data were then assigned to each representative sequence using the Ribosomal Database Project classifier. The differences in the dominant species in different groups, multiple sequence alignment were conducted using the PyNAST software to study phylogenetic relationship of different OTUs. OTU abundance information was normalized using a standard of sequence number corresponding to the sample with the least sequences. Alpha diversity is applied in analyzing complexity of species diversity for a sample through 4 indices, including Chao1, Shannon, Simpson, and Observed species. All these indices in our samples were calculated with QIIME (version 1.8.0). Beta‐diversity analysis was used to evaluate differences in samples in species complexity. Beta diversity was calculated by principle co‐ordinate analysis (PCoA) and cluster analysis by QIIME software (version 1.8.0).

### 2.4 Statistical analysis

Analysis was performed using SPSS version 21.0 software for Windows (SPSS Inc.). All data were reported as mean ± standard deviations (*SD*). Data with a normal distribution were compared with the independent sample use *t* test. Repeated measurement data were analyzed by analysis of variance. Statistical analysis of ordered grade data was weighted on the basis of the number and conducted by the Mann–Whitney test. Differences in taxa communities of intestinal microbiota among samples were compared by the UniFrac distance distribution. The Kruskal–Wallis test was used to identify genera with significant differences among groups. The level of statistical significance was set at *p* < 0.05.

## 3 RESULTS

### 3.1 Jlus66 effectively decreased the oxidative stress, inflammatory cytokines, and LPS molecules

Twenty weeks of HFD induction significantly increased the MDA levels but decreased the SOD and GSH‐Px activities in serum (*p* < 0.001).The MDA levels of all three Jlus66‐treated groups were markedly lower than that of the HFD group in serum. Compared to the HFD group rats, the SOD and GSH‐Px levels of ProL, ProM, and ProH groups significantly increased (*p* < 0.05) in serum (Table 1). Though the activities of SOD in three Pro groups and the GSH‐Px in ProL and ProM groups in liver were not significantly changed, there was slight improvement on average (Table 1). Interestingly, all data showed a dose–response relationship, which indicated that the beneficial effect of JLus66 depended on its quantity and activity.

**Table 1:** Levels of SOD, GSH‐Px, MDA, LPS, TNF‐α, IL‐6, and IL‐10 in serum or in liver

Biochemical indicators	Groups				
	Con	HFD	ProL	ProM	ProH
MDA^a^ (mmol/L)	11.35 ± 0.95	14.87 ± 1.13*	2.50 ± 0.65	11.31 ± 0.33^†^	10.16 ± 0.31^†^
SOD^a^ (U/ml)	210.81 ± 13.15	178.48 ± 17.28*	207.24 ± 6.89^‡^	211.84 ± 10.94^†^	230.34 ± 7.56^†^
SH‐Px^a^ (U/ml)	243.91 ± 8.23	198.54 ± 11.56*	227.10 ± 18.16^‡^	241.84 ± 13.77^†^	244.52 ± 14.45^†^
LPS^a^ (EU/ml)	0.23 ± 0.01	0.67 ± 0.01*	0.47 ± 0.00	0.40 ± 0.01^†^	0.39 ± 0.01^†^
TNF‐α^a^ (pg/ml)	68.36 ± 12.81	187.08 ± 36.41*	152.19 ± 35.88^‡^	149.2 ± 38.42^‡^	144.32 ± 35.29^†^
IL−6^a^ (pg/ml)	31.75 ± 9.83	88.43 ± 19.2*	74.11 ± 16.47^‡^	65.79 ± 24.83^‡^	62.51 ± 19.85^†^
IL−10^a^ (pg/ml)	114.74 ± 16.55	72.87 ± 15.45*	83.59 ± 13.86	90.31 ± 16.14^‡^	89.26 ± 16.45^†^
MDA^b^ (mmol/L)	1.36 ± 0.27	2.96 ± 0.35*	2.50 ± 0.65	2.03 ± 0.65^†^	1.28 ± 0.17^†^
SOD^b^ (U/ml)	363.61 ± 26.71	247.98 ± 28.89*	282.56 ± 31.42	291.21 ± 26.65	300.41 ± 18.07
SH‐Px^b^ (U/ml)	390.46 ± 37.22	232.78 ± 27.70**	269.27 ± 36.85	306.69 ± 30.76	375.11 ± 25.20^†^

a Mean in serum.

b Means in serum; *n* = 8.

* *p* < 0.01 or

** *p* < 0.05 compared to Con.

^†^ *p* < 0.01 or

^‡^ *p* < 0.05 compared to HFD.

The level of LPS in serum of the HFD group was remarkably higher than that of the Con group (*p* < 0.001). However, Jlus66 administration distinctly decreased the LPS levels (*p* < 0.01 for the ProM and ProH groups; Table 1). HFD induction significantly raised the activity of proinflammatory factors (TNF‐α and IL‐6) in serum, but lowered the anti‐inflammatory factor (IL‐10) compared to the Con group (*p* < 0.001). Jlus66 treatment reduced the activity of TNF‐α and IL‐6, inversely enhanced the activity of IL‐10 compared to the HFD group (*p* < 0.05 or *p* < 0.01; Table 1).

### 3.2 Jlus66 regulated the composition of intestinal flora

Alpha diversity (species richness) was accessed based on OTUs of each sample. Four indices (Observed species, Chao1, Shannon, and Simpson) were used to analyze alpha diversity. The Chao1 and Observed species mainly reflect the number of OTU species in the sample. The Shannon and Simpson reflect the number of species and the average or uniformity of abundance of different species in the sample. The HFD group had significantly lower alpha‐diversity indexes (Observed species, 824.67 ± 74.17; Chao1, 1,295 ± 129.89; Shannon, 5.79 ± 0.50; and Simpson, 0.90 ± 0.03) than that of the control group. The four indexes of the ProH group significantly increased compared with the HFD group (*p* < 0.01 or *p* < 0.05, Table 2).

**Table 2:** Numerical values of four alpha‐diversity indexes

Group	Observed species	Chao1	Shannon	Simpson
HFD	824.67 ± 74.17*	1,295 ± 129.89*	5.79 ± 0.50**	0.90 ± 0.03**
Control	1,376.33 ± 117.98	1885.15 ± 157.56	8.39 ± 0.24	0.99 ± 0
ProH	1,420.67 ± 233.82^†^	1950.92 ± 259.51^‡^	8.48 ± 0.46^‡^	0.99 ± 0.01^‡^

* *p* < 0.01 or

** *p* < 0.05 compared to Con.

^†^ *p* < 0.01 or

^‡^ *p* < 0.05 compared to HFD; *n* = 6.

**Figure 1:**
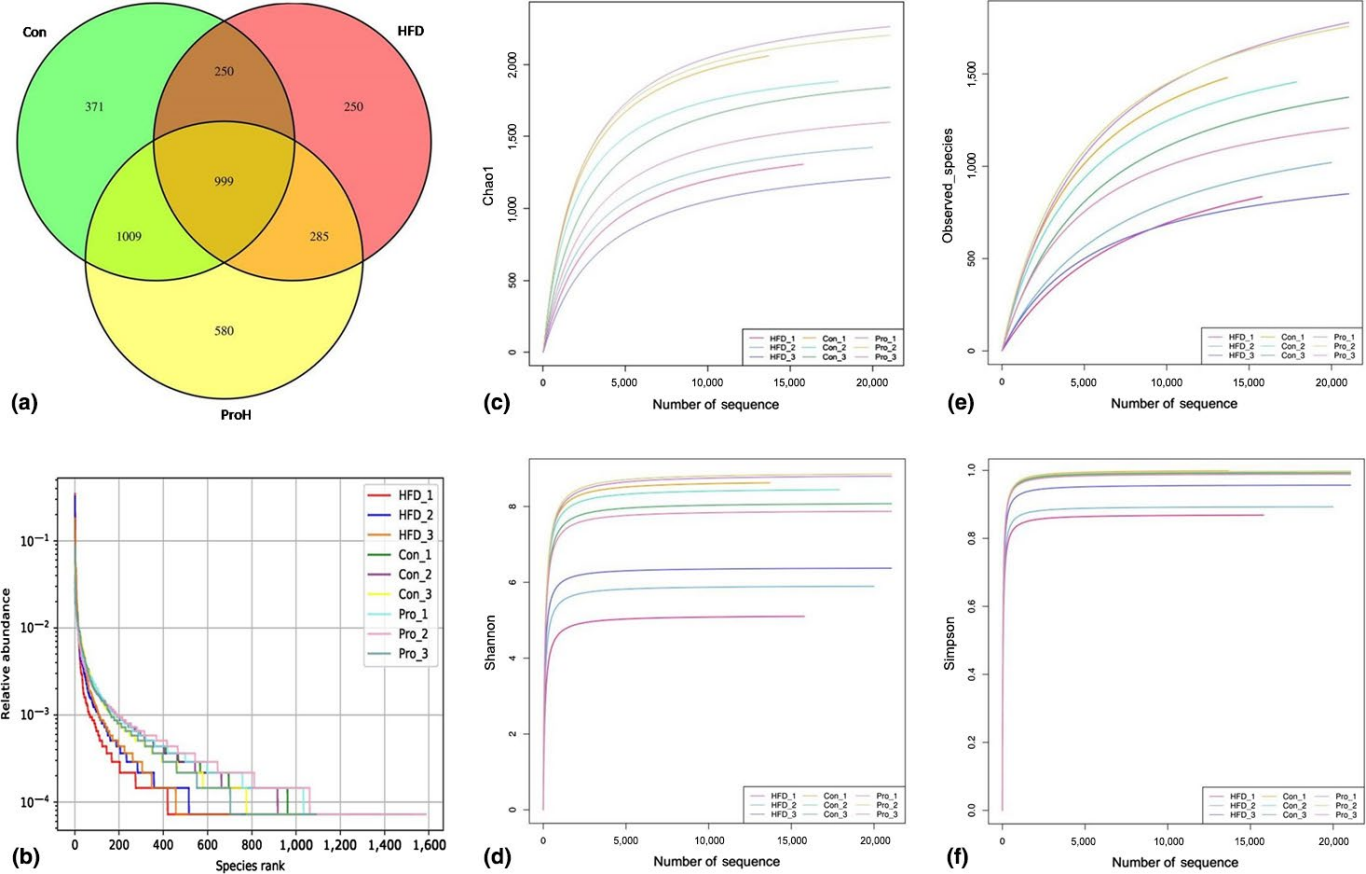
Alpha diversity. (a) Venn Diagram: The Venn diagram intuitively showed the common or unique OTUs among Con, HFD, and ProH samples; (b) Rank Abundance Graph: Different color curves represent different samples. The abscissa is the OUT abundance level, and the ordinate is the relative abundance of OUT. The steepness of the curve expresses the difference between samples in OUT abundance. (c, d, e, and f) Rarefaction curves of Chao1, Shannon, Observed species, and Simpson: Observed species and Chao 1 indicate sample species richness; Shannon and Simpson indicate sample species diversity

**Figure 2:**
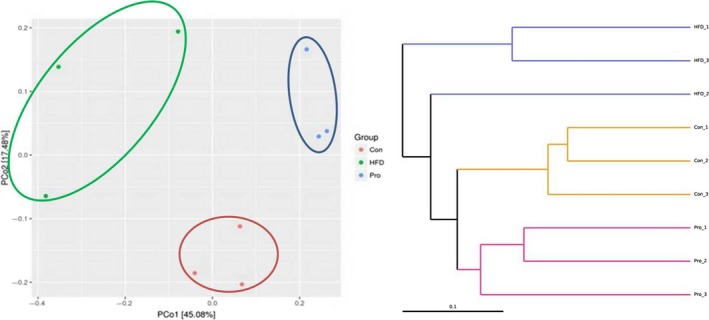
Beta diversity. Left, PCoA plot with weighted UniFrac matrices: The distance between samples indicates the similarity of microbial composition between samples. Right, UPGMA hierarchical clustering analysis: The different colors of branches represent different groups. Clustering tree shows the similarity between samples. The shorter the branch length between samples, the more similar the two simples are

The Venn diagram intuitively showed the common or unique OTUs among different samples (Figure 1a). As shown in Figure 1a, the common OTUs between Con and HFD, Con and ProH, ProH and HFD were 1,249, 2,008, and 1,284, respectively. The ProH group had the highest unique OTUs (580) which was twofold over the HFD group (250), and even greater than the Con group (371). The result reflected that HFD decreased the diversity of intestinal flora, while JLus66 restored the abundance of intestinal flora. The grade abundance curve visually describes the species richness and species uniformity of different samples (Figure 1b). In the horizontal direction, the width of the curve reflects the richness of the species. The larger the range of the curve on the horizontal axis, the more the number of species (the number of OTUs ordered). In the vertical direction, the steeper the gradient of the curve, the lower the abundance of the high‐level specie is, indicating biased species distribution. Principal co‐ordinate analysis (PCoA) and unweighted pair group method with arithmetic mean(UPGMA) also clearly showed that the intestinal flora composition of JLus66‐treated rats was more similar to the control rats but different from the HDF group (Figure 2).

**Figure 3:**
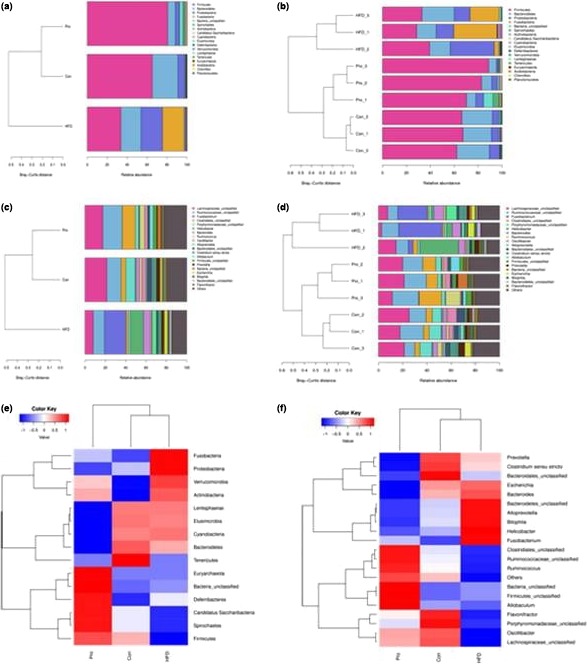
The relative abundance of intestinal flora. (a and b), The top 20 species relative abundance distribution map at phylum level of each sample. (c and d), The top 20 species relative abundance distribution map at genus level of each sample. (e) Taxa heatmap at phylum level; (f) taxa heatmap at genus level. In heatmap, the gradient from blue to red reflects the change from low to high

The relative abundances of different taxa between different samples were detected with cluster analysis and heatmap (Figure 3). In the heatmap, the greater the abundance of a species, the darker the color is. The row represents abundance of each family in different samples, and the column shows the top 20 families of each sample.

At phylum level, eighteen phyla were detected and five of them displayed significant difference between the HFD and Con or ProH group. Firmicutes, Bacteroidetes Proteobacteria, and Fusobacteria were the dominant phylums in these samples. HFD significantly decreased Firmicutes (33.7% vs. 65.3%, *p* < 0.05) but increased abundances of Bacteroidetes, Proteobacteria, and Fusobacteria (20.22% vs. 25.58%, 6.64% vs. 21.59%, 0.01% vs. 21.43%, *p* < 0.05) compared to the control group. JLus66 treatment significantly increased Firmicutes (80.6%, *p* < 0.05) and decreased Proteobacteria and Fusobacteria (4.13% and 0.09%). The decrease in Bacteroidetes in the ProH group (7.7% vs. 20.2%, *p* < 0.05) led to an increase in Firmicutes/Bacteroidetes ratio between the ProH and HFD groups (10.47 vs. 1.32) (Table 3).

**Table 3:** The relative abundance of intestinal flora at phylum, family, and genus levels

Flora	Con	HFD	ProH
Phylum			
Firmicutes (G+)	65.27 ± 2.81	33.66 ± 5.42*	80.60 ± 9.45**
Bacteroidetes (G−)	20.22 ± 5.91	25.58 ± 1.76*	7.70 ± 1.04**
Proteobacteria (G−)	6.64 ± 1.64	21.59 ± 13.25*	4.13 ± 2.41**
Fusobacteria (G−)	0.01 ± 0.00	21.43 ± 16.37*	0.09 ± 0.15**
Candidatus Saccharibacteria (G−)	0.22 ± 0.20	0.06 ± 0.05	1.31 ± 0.12**
Family			
Ruminococcaceae (G+)	23.80 ± 6.84	13.74 ± 2.22*	30.11 ± 6.45**
Lachnospiraceae (G+)	28.57 ± 3.60	11.57 ± 4.72*	21.14 ± 5.33
Fusobacteriaceae (G−)	0.01 ± 0.00	21.43 ± 16.37*	0.09 ± 0.15**
Clostridiales_unclassified (G−)	4.61 ± 0.45	2.24 ± 1.39*	12.88 ± 4.99**
Bacteroidaceae (G−)	3.45 ± 0.41	6.88 ± 1.66*	0.38 ± 0.21**
Porphyromonadaceae (G+)	10.09 ± 1.28	2.73 ± 0.42*	3.82 ± 1.27
Helicobacteraceae (G−)	0.94 ± 0.30	12.93 ± 16.65*	0.43 ± 0.51**
Enterobacteriaceae (G−)	2.25 ± 3.54	4.94 ± 3.77	0.15 ± 0.04**
Clostridiaceae (G−)	0.20 ± 0.34	0.17 ± 0.07	1.86 ± 2.49**
Eubacteriaceae (G+)	0.03 ± 0.01	0.21* ± 0.20	0.03 ± 0.00**
Peptostreptococcaceae (G+)	0.37 ± 0.28	0.04 ± 0.05*	1.54 ± 1.20**
Oscillospiraceae (G−)	0.06 ± 0.03	0.01 ± 0.01	0.13 ± 0.06**
Lactobacillaceae (G+)	0.61 ± 0.48	0.03 ± 0.01*	1.59 ± 1.41**
Bifidobacteriaceae (G+)	0.01 ± 0.00	0.01 ± 0.00	0.12 ± 0.01**
Genus			
*Bacteroides* (G−)	3.45 ± 0.41	6.82 ± 1.62*	0.35 ± 0.22**
*Fusobacterium* (G−)	0.01 ± 0.00	21.42 ± 16.37*	0.09 ± 0.15**
*Bifidobacterium* (G+)	0.01 ± 0.00	0.01 ± 0.01	0.12 ± 0.08**
*Escherichia* (G−)	1.86 ± 2.93	3.01 ± 2.47	0.04 ± 0.01**
*Blautia* (G+)	0.13 ± 0.21	0.68* ± 0.12	0.18 ± 0.25**
*Roseburia* (G+)	2.74 ± 1.98	0.09 ± 0.12*	0.54 ± 0.28**
*Lachnospiraceaincertaesedis* (G+)	1.01 ± 0.79	0.00 ± 0.00*	0.24 ± 0.06**
*Ruminococcus* (G+)	2.24 ± 1.21	0.92 ± 0.70	5.09 ± 6.05
*Ruminococcus2* (G+)	0.25 ± 0.20	0.04 ± 0.02*	0.21 ± 0.06**
*Oscillibacter* (G−)	4.12 ± 3.04	0.68 ± 0.47*	2.95 ± 1.80
*Lactobacillus* (G+)	0.61 ± 0.48	0.03 ± 0.01*	1.59 ± 1.41**
*Collinsella* (G−)	0 ± 0.00	0.09 ± 0.11*	0.02 ± 0.01

* *p* < 0.05 compared to Con.

** *p* < 0.05 compared to HFD, (G+) represents Gram‐positive, (G−) represents Gram‐negative, *n* = 6.

At family level, six families (Ruminococcaceae, Lachnospiraceae, Clostridiales, Porphyromonadaceae, Peptostreptococcaceae, and Lactobacillaceae) showed lower abundance and four families (Fusobacteriaceae, Bacteroidaceae, Helicobacteraceae, and Eubacteriaceae) showed higher abundance in the HFD group (*p* < 0.05) than that in the Con group. JLus66 administration obviously recovered the flora structure at family level compared to the HFD group (*p* < 0.05). It is noteworthy that the quantity of Clostridiaceae, Oscillospiraceae, and Bifidobacteriaceae was discovered to be increased in the JLus66‐treated group. Enterobacteriaceae which is an important family increased to 4.94% (in HFD) and obviously declined to 0.15% (in Pro, *p* < 0.05) (Table 3).

The reduction of Firmicutes in the HFD group was probably because of the decreasing abundance of Lachnospiraceae and Ruminococcaceae families. The most prominent changes at genus level were observed within the two families. Most of the genera within Lachnospiraceae family exhibited a similar trend, such as *Lachnospiraceaincertaesedis* and *Roseburia*. Expectedly, the three above genera in the ProH group achieved an opposite effect (Table 3). Two members of Ruminococcaceae family, *Oscillibacter* and *Ruminococcus,* were discovered to be at very lower abundance in the HFD group than over 6‐fold abundance in the control group (*p* < 0.05), whereas JLus66 almost restored their abundances (*p* > 0.05). Fusobacteriaceae and *Fusobacterium* as genera of Fusobacteria were all coincided with the increase in Fusobacteria (Table 3; Figure 3c,d). Similarly, the increased abundance of Proteobacteria depended on the increased abundance of Enterobacteriaceae and *Escherichia* (of family Enterobacteriaceae) (Table 3; Figure 3d,f).

## 4 DISCUSSION

According to our previous study, JLus66 effectively improved NAFLD by reducing of lipid accumulation (Ye et al., 2017). Now that OxS plays a key role in the course of NAFLD (Wu et al., 2018). Therefore, in the present study, we aim to explore the protective effect of JLus66 on oxidative damages in HFD‐induced rats. As one of the end products of lipid peroxidation, MDA levels reflect the degree of peroxidation of membrane lipids (Foyer & Noctor, 2009). SOD is a main antioxidant enzyme, which can protect tissues from oxidative damages (Shih, Wu, & Lin, 2005). HFD feeding remarkably increased the level of MDA in serum and liver, on the contrary, decreased SOD and GSH‐Px activities, suggesting a decrease in antioxidant defense function. Treatment with 1, 2, 4×10^10 ^cfu JLus66 daily for twenty weeks enhanced the antioxidant defenses. The SOD activity increased 29.1% in serum. Similarly, the GSH‐Px activity increased 23.2% in serum and 61.1% in liver. Meanwhile, JLus66 reduced MDA concentrations in serum as well as in liver (Table 1). Our results clearly confirmed that JLus66 can protect rats from HFD‐induced oxidative damages.

Cytokines are associated with immune response, inflammation, and tissue damage or repair. Intestinal bacteria contribute to activating relevant mechanisms and subsequently triggering an inflammatory adaptive immune response that involves several cytokines, such as interleukin‐1, interleukin‐2, IL‐6, IL‐10, interleukin‐22, and TNF‐α (Decicco, Rikans, Tutor, & Hornbrook, 1998). Our results showed that HFD induction increased the concentration of LPS, accompanied with the increase in TNF‐α and IL‐6 in serum, as well as disordered the intestinal flora by increasing abundance of Gram‐negative flora (Bacteroidetes, Proteobacteria, and Fusobacteria) and decreasing abundance of gram‐negative flora (Firmicutes). Lots of previous research indicated that LPS plays an important role between intestinal flora and low‐grade inflammation involved in metabolic diseases. Dysbiosis can increase intestinal permeability and then induce low‐grade chronic inflammation by transporting bacterial LPS into plasma (Ritze et al., 2014). Specifically, the bacterial LPS largely abundant in enteric gram‐negative flora can trigger the inflammatory processes in NAFLD (Cani et al., 2007; Musso, Gambino, & Cassader, 2010; Peverill, Powell, & Skoien, 2014). IL‐6 is an effective activator of hepatic signal transducers and activators of transcription 3 (STAT3) pathway and is diffusely featured as a participator in different facets of liver pathophysiology. TNF‐α as a proinflammatory cytokine can stimulate NFκB pathways and cause damage to cells (Bian et al., 2017). Treatment with JLus66 increased the abundance of Gram‐positive flora (Firmicutes) and decreased Gram‐negative flora (Bacteroidetes, Proteobacteria, and Fusobacteria), recovering the Gram‐positive‐to‐Gram‐negative ratio, which result in decrease in LPS, accompanied with decrease in TNF‐α and IL‐6 activities in serum, and then lightened inflammation in hepatic tissues. In conclusion, JLus66 can recover the composition of gut microbiota by increasing the flora ratio of Gram‐positive to Gram‐negative, which contributes to the reduction of inflammation.

As shown in Table 3, JLus66 increased many beneficial bacteria communities, such as Lactobacillus and Bifidobacterium, among which many strains are used as probiotics. Lactobacillus and Bifidobacterium have been demonstrated to suppress hepatic inflammation in mice and humans (Li et al., 2003), and reduce hepatic fibrosis (Okubo et al., 2013). Bifidobacteria are also known to be able to ferment guar gum with active bile salt hydrolases (Noack, Kleessen, Proll, Dongowski, & Blaut, 1998; Ohashi et al., 2015). Since unconjugated bile acids are less efficient in solubilization and absorption of fecal lipids (Begley, Hill, & Gahan, 2006), Bifidobacterium may be responsible for the elevated fecal lipid excretion by increasing bile salt hydrolase activity and unconjugated bile acids (Janssen et al., 2017). Importantly, Bifidobacterium has been shown to reduce intestinal endotoxin levels and improve mucosal barrier function in rodents (Griffiths et al., 2004; Wang et al., 2006). Interestingly, JLus66 increased over 3‐fold and 12‐fold abundance of *Lactobacilli and Bifidobacterium* in the ProH group, which may contribute to the reduction of TG, TC, and LDL levels in liver in our previous study (Ye et al., 2017), and the lighten inflammation of liver in the current study.

The relationship between short‐chain fatty acids (SCFAs) and NAFLD has drawn many scholars concerning in recent years. HFD can change the balance of SCFAs by decreasing formation of butyrate and increasing acetate, which facilitates the development of NAFLD (Jakobsdottir, Xu, Molin, Ahrné, & Nyman, 2013; Jun, Cathrin, Anna Janina, Doreen, & Ina, 2015). The gut microbiota impact the host energetic balance by fermenting resistant starch and nonstarch polysaccharides to SCFAs (mainly acetate, propionate, and butyrate) (Topping & Clifton, 2001). Butyrate and propionate can regulate intestinal physiology and immune function, while acetate acts as a substrate for lipogenesis and gluconeogenesis (Macfarlane & Macfarlane, 2011). Our study showed a significant increase in relative abundance of Ruminococcus and Roseburia in the ProH group. Notably, Ruminococcus and Roseburia are butyrate producers. Candida is able to degrade starches, liberating sugars to be fermented by *Prevotella* (phylum Bacteroidetes) and Ruminococcus species, and thus increase energy production from food in the gut and reduce the energy available for absorption and utilization (Stams & Plugge, 2009). Many previous studies indicated that NAFLD is associated with a lower proportion of the Ruminococcaceae family of the phylum Firmicutes (Jiang et al., 2015; Mouzaki et al., 2013; Pataky et al., 2016). Indeed, a study in humans showed the favorable metabolic effects of fecal transplantation from lean donors into patients with obesity with a marked increase in the proportion of the butyrate producer *Roseburiaintestinalis* (Vrieze et al., 2012). Also, compared to those without NAFLD, patients with NAFLD had higher abundance of Bacteroides and lower abundance of Prevotella (Boursier et al., 2016). Thus, in HFD feeding rats, the Firmicutes/Bacteroidetes ratio was increased by Jlus66, which was also related to increased levels of the beneficial SCFA butyrate, and decreased body weight, adiposity, and hepatic triglycerides (Cowan et al., 2014).

In addition, many bacteria can promote NAFLD through alcohol production. Such *Escherichia* and other Enterobacteriaceae, which are alcohol producers, were found to be substantially increased in patients with NAFLD (Zhu et al., 2013). Ethanol produced in the gut may have direct toxic effects in the liver, simultaneously, increasing intestinal permeability and portal LPS levels, triggering Toll‐like receptor and inflammasome activation (Parlesak, Schäfer, Schütz, Bode, & Bode, 2000). JLus66 dropped the proportion of Enterobacteriaceae (at family level) to 0.15% and *Escherichia* (at genus level) to 0.04%, which were far lower than 4.94% and 3.01% in the HFD group, respectively. These results lend further support useful effect of JLus66 on NAFLD.

## 5 CONCLUSION

According to the current study, we concluded that Jlus66 can reduce OxS and inflammation via regulation of intestinal flora in HFD‐induced NAFLD. All results further proved the potential of JLus66 as probiotics. Our study also supports the assumption that probiotics may be an ideal method for controlling NAFLD. Further research is necessary to clarify the relevance of oxidative stress, inflammation, and the gut microbial taxa discovered in our work to the pathogenesis and progression of NAFLD.

## CONFLICTS OF INTEREST

The authors have declared no conflicts of interest.

## ETHICAL STATEMENT

All animal experiments were conducted in accordance with the Directive 2010/63/EU and approved by the Institutional Animal Care and Use Committee of Jilin University, IACUC (Approve number: 20180101).

## AUTHORS' CONTRIBUTION

T. H. Zhang conceived this research; W. Wang and H. Q. Ye designed the experiments; W. Wang, H. Q. Ye, Q. Li, H. Q. Liu, W. H. Chai, and X. Y. Wang performed the research; H. Q. Ye, C. Y. Sun, and Y. Yuan analyzed the data; H. Q. Ye, and C. H. Zhao prepared the paper.

## References

[fsn31118-bib-0001] Begley, M. , Hill, C. , & Gahan, C. G. M. (2006). Bile salt hydrolase activity in probiotics. Applied & Environmental Microbiology, 72(3), 1729–1738. 10.1128/AEM.72.3.1729-1738.2006 16517616 PMC1393245

[fsn31118-bib-0002] Bellentani, S. , Scaglioni, F. , Marino, M. , & Bedogni, G. (2010). Epidemiology of non‐alcoholicfatty liver disease. Digestive Diseases, 28(1), 155–161. 10.1159/000282080 20460905

[fsn31118-bib-0003] Bian, X. , Tu, P. , Chi, L. , Gao, B. , Ru, H. , & Lu, K. (2017). Saccharin induced liver inflammation in mice by altering the gut microbiota and its metabolic functions. Food and Chemical Toxicology, 107, 530–539. 10.1016/j.fct.2017.04.045 28472674 PMC5647777

[fsn31118-bib-0004] Borrelli, A. , Bonelli, P. , Tuccillo, F. M. , Goldfine, I. D. , Evans, J. L. , Buonaguro, F. M. , & Mansini, A. (2018). Role of gut microbiota and oxidative stress in the progression of non‐alcoholic fatty liver disease to hepatocarcinoma: Current and innovative therapeutic approaches. Redox Biology, 15, 467–479. 10.1016/j.redox.2018.01.009 29413959 PMC5975181

[fsn31118-bib-0005] Boursier, J. , Mueller, O. , Barret, M. , Machado, M. , Fizanne, L. , Araujo‐Perez, F. , … Diehl, A. M. (2016). The severity of nonalcoholic fatty liver disease is associated with gut dysbiosis and shift in the metabolic function of the gut microbiota. Hepatology, 63(3), 764–775. 10.1002/hep.28356 26600078 PMC4975935

[fsn31118-bib-0006] Cani, P. D. , Amar, J. , Iglesias, M. A. , Poggi, M. , Knauf, C. , Bastelica, D. , … Burcelin, R. (2007). Metabolic endotoxemia initiates obesity and insulin resistance. Diabetes, 56(7), 1761–1772. 10.2337/db06-1491 17456850

[fsn31118-bib-0007] Chen, Z. , Liu, F. , Zheng, N. , Guo, M. , Bao, L. , Zhan, Y. , … Ding, G. (2019). Wuzhi capsule (Schisandra Sphenanthera extract) attenuates liver steatosis and inflammation during non‐alcoholic fatty liver disease development. Biomedicine & Pharmacotherapy, 110, 285–293. 10.1016/j.biopha.2018.11.069 30522014

[fsn31118-bib-0008] Cowan, T. E. , Palmnäs, M. S. A. , Yang, J. , Bomhof, M. R. , Ardell, K. L. , Reimer, R. A. , … Shearer, J. (2014). Chronic coffee consumption in the diet‐induced obese rat: Impact on gut microbiota and serum metabolomics. Journal of Nutretion Biochemistry, 25(4), 489–495. 10.1016/j.jnutbio.2013.12.009 24629912

[fsn31118-bib-0009] Decicco, L. , Rikans, L. , Tutor, C. , & Hornbrook, K. (1998). Serum and liver concentrations of tumor necrosis factor alpha and interleukin‐1beta following administration of carbon tetrachloride to male rats. Toxicology Letters, 98(1–2), 115–121. 10.1016/S0378-4274(98)00110-6 https://doi.org/ 9776568

[fsn31118-bib-0010] El Hadi, H. , Vettor, R. , & Rossato, M. (2018). Vitamin E as a Treatment for Nonalcoholic Fatty Liver Disease: Reality or Myth? Antioxidants, 7(1), 10.3390/antiox7010012 PMC5789322 29337849

[fsn31118-bib-0011] Erhardt, A. , Stahl, W. , Sies, H. , Lirussi, F. , Donner, A. , & Häussinger, D. (2011). Plasma levels of vitamin E and carotenoids are decreased in patients with nonalcoholic steatohepatitis (NASH). European Journal of Medical Research, 16(2), 76–78. 10.1186/2047-783X-16-2-76 21463986 PMC3353426

[fsn31118-bib-0012] Esposito, E. , Iacono, A. , Bianco, G. , Autore, G. , Cuzzocrea, S. , Vajro, P. , Meli, R. (2009). Probiotics Reduce the inflammatory response induced by a high‐fat diet in the liver of young rats. Journal of Nutition, 139(5), 905–911. 10.3945/jn.108.101808 19321579

[fsn31118-bib-0014] Fadrosh, D. W. , Ma, B. , Gajer, P. , Sengamalay, N. , Ott, S. , Brotman, R. M. , & Ravel, J. (2014). An improved dual‐indexing approach for multiplexed 16S rRNA gene sequencing on the Illumina MiSeq platform. Microbiome, 2, 6 https://doi.10.1186/2049-2618-2-6 24558975 10.1186/2049-2618-2-6 PMC3940169

[fsn31118-bib-0015] Foyer, C. H. , & Noctor, G. (2009). Redox regulation in photosynthetic organisms: Signaling, acclimation, and practical implications. Antioxidants & Redox Signaling, 11(4), 861–905. 10.1089/ars.2008.2177 19239350

[fsn31118-bib-0016] Griffiths, E. A. , Duffy, L. C. , Schanbacher, F. L. , Qiao, H. , Dryja, D. , Leavens, A. , … Ogra, P. L. (2004). In vivo effects of bifidobacteria and lactoferrin on gut endotoxin concentration and mucosal immunity in Balb/c mice. Digestive Disease and Science, 49(4), 579–589. 10.1023/B:DDAS.0000026302.92898.ae 15185861

[fsn31118-bib-0017] Guarner, F. , & Malagelada, J. R. (2003). Role of bacteria in experimental colitis. Best Practice & Research in Clinical Gastroenterology, 17(5), 793–804. 10.1016/S1521-6918(03)00068-4 14507589

[fsn31118-bib-0019] Jakobsdottir, G. , Xu, J. , Molin, G. , Ahrné, S. , & Nyman, M. (2013). High‐fat diet reduces the formation of butyrate, but increases succinate, inflammation, liver fat and cholesterol in rats, while dietary fibre counteracts these effects. PLoS ONE, 8(11), e80476 10.1371/journal.pone.0080476 24236183 PMC3827442

[fsn31118-bib-0020] Janssen, A. W. F. , Houben, T. , Katiraei, S. , Dijk, W. , Boutens, L. , van der Bolt, N. , … Kersten, S. (2017). Modulation of the gut microbiota impacts nonalcoholic fatty liver disease: A potential role for bile acids. Journal of Lipid Research, 58(7), 1399–1416. 10.1194/jlr.M075713 28533304 PMC5496037

[fsn31118-bib-0021] Jiang, W. , Wu, N. A. , Wang, X. , Chi, Y. , Zhang, Y. , Qiu, X. , … Liu, Y. (2015). Dysbiosis gut microbiota associated with inflammation and impaired mucosal immune function in intestine of humans with non‐alcoholic fatty liver disease. Scientific Reports, 5, 8096 10.1038/srep08096 25644696 PMC4314632

[fsn31118-bib-0022] Jun, J. C. , Cathrin, S. , Anna Janina, E. , Doreen, Z. , & Ina, B. (2015). Supplementation of sodium butyrate protects mice from the development of non‐alcoholic steatohepatitis (NASH). British Journal of Nutrition, 114(11), 1745–1755. 10.1017/S0007114515003621 26450277

[fsn31118-bib-0023] Li, Z. , Yang, S. , Lin, H. , Huang, J. , Watkins, P. A. , Moser, A. B. , … Diehl, A. M. (2003). Probiotics and antibodies to TNF inhibit inflammatory activity and improve nonalcoholic fatty liver disease. Hepatology, 37(2), 343–350. 10.1053/jhep.2003.50048 12540784

[fsn31118-bib-0024] Macfarlane, G. T. , & Macfarlane, S. (2011). Fermentation in the human large intestine: Its physiologic consequences and the potential contribution of prebiotics. Journal of Clinical Gastroenterology, 45(S), S120–S127. 10.1097/MCG.0b013e31822fecfe 21992950

[fsn31118-bib-0025] Marchesi, J. R. , Adams, D. H. , Fava, F. , Hermes, G. D. A. , Hirschfield, G. M. , Hold, G. , … Hart, A. (2016). The gut microbiota and host health: A new clinical frontier. Gut, 65(2), 330–339. 10.1136/gutjnl-2015-309990 26338727 PMC4752653

[fsn31118-bib-0026] Martin, F. , Verdu, E. , Wang, Y. , Dumas, M. , Yap, I. , Cloarec, O. , … Nicholson, J. K. (2006). Transgenomic metabolic interactions in a mouse disease model: Interactions of trichinella spiralis infection with dietary lactobacillus paracasei supplementation. Journal of Proteome Research, 5(9), 2185–2193. 10.1021/pr060157b 16944930

[fsn31118-bib-0027] Masarone, M. , Rosato, V. , Dallio, M. , Gravina, A. G. , Aglitti, A. , Loguercio, C. , … Persico, M. (2018). Role of oxidative stress in pathophysiology of nonalcoholic fatty liver disease. Oxidative Medicine and Cellular Longevity, 2018(3), 1–14. 10.1155/2018/9547613 PMC6016172 29991976

[fsn31118-bib-0028] Miele, L. , Valenza, V. , La Torre, G. , Montalto, M. , Cammarota, G. , Ricci, R. , … Grieco, A. (2009). Increased intestinal permeability and tight junction alterations in nonalcoholic fatty liver disease. Hepatology, 49(6), 1877–1887. 10.1002/hep.22848 19291785

[fsn31118-bib-0029] Mouzaki, M. , Comelli, E. M. , Arendt, B. M. , Bonengel, J. , Fung, S. K. , Fischer, S. E. , … Allard, J. P. (2013). Intestinal microbiota in patients with nonalcoholic fatty liver disease. Hepatology, 58(1), 120–127. 10.1002/hep.26319 23401313

[fsn31118-bib-0030] Musso, G. , Gambino, R. , & Cassader, M. (2010). Gut microbiota as a regulator of energy homeostasis and ectopic fat deposition: Mechanisms and implications for metabolic disorders. Current Opinion in Lipidology, 21(1), 76–83. 10.1097/MOL.0b013e3283347ebb 19915460

[fsn31118-bib-0031] Noack, J. , Kleessen, B. , Proll, J. , Dongowski, G. , & Blaut, M. (1998). Dietary guar gum and pectin stimulate intestinal microbial polyamine synthesis in rats. Journal of Nutrition, 128(8), 1385–1391. 10.1093/jn/128.8.1385 9687560

[fsn31118-bib-0032] Ohashi, Y. , Sumitani, K. , Tokunaga, M. , Ishihara, N. , Okubo, T. , & Fujisawa, T. (2015). Consumption of partially hydrolysed guar gum stimulates bifidobacteria and butyrate‐producing bacteria in the human large intestine. Beneficial Microbes, 6(4), 451–455. 10.3920/BM2014.0118 25519526

[fsn31118-bib-0033] Okubo, H. , Sakoda, H. , Kushiyama, A. , Fujishiro, M. , Nakatsu, Y. , Fukushima, T. , … Asano, T. (2013). Lactobacillus casei strain Shirota protects against nonalcoholic steatohepatitis development in a rodent model. America Journal of Physiology Gastrointestinal and Liver Physiology, 305(12), G911–918. 10.1152/ajpgi.00225.2013 24113768

[fsn31118-bib-0034] Parlesak, A. , Schäfer, C. , Schütz, T. , Bode, J. C. , & Bode, C. (2000). Increased intestinal permeability to macromolecules and endotoxemia in patients with chronic alcohol abuse in different stages of alcohol‐induced liver disease. Journal of Hepatology, 32(5), 742–747. 10.1016/S0168-8278(00)80242-1 10845660

[fsn31118-bib-0036] Pataky, Z. , Genton, L. , Spahr, L. , Lazarevic, V. , Terraz, S. , Gaïa, N. , … Pichard, C. (2016). Impact of hypocaloric hyperproteic diet on gut microbiota in overweight or obese patients with nonalcoholic fatty liver disease: A pilot study. Digestive Diseases and Science, 61(9), 2721–2731. 10.1007/s10620-016-4179-1 27142672

[fsn31118-bib-0037] Peverill, W. , Powell, L. W. , & Skoien, R. (2014). Evolving concepts in the pathogenesis of NASH: Beyond steatosis and inflammation. International Journal of Molecular Sciences, 15(5), 8591–8638. 10.3390/ijms15058591 24830559 PMC4057750

[fsn31118-bib-0038] Ritze, Y. , Bardos, G. , Claus, A. , Ehrmann, V. , Bergheim, I. , Schwiertz, A. , … Bischoff, S. C. (2014). Lactobacillus rhamnosus GG protects against non‐alcoholic fatty liver disease in mice. PLoS ONE, 9(1), e80169 10.1371/journal.pone.0080169 24475018 PMC3903470

[fsn31118-bib-0039] Rotman, Y. , & Sanyal, A. J. (2017). Current and upcoming pharmacotherapy for non‐alcoholic fatty liver disease. Gut, 66(1), 180–190. 10.1136/gutjnl-2016-312431 27646933

[fsn31118-bib-0040] Shih, C. C. , Wu, Y. W. , & Lin, W. C. (2005). Aqueous extract of Anoectochilus formosanus attenuate hepatic fibrosis induced by carbon tetrachloride in rats. Phytomedicine, 12(6–7), 453–460. 10.1016/j.phymed.2004.02.008 16008122

[fsn31118-bib-0041] Spahis, S. , Delvin, E. , Borys, J. M. , & Levy, E. (2017). Oxidative stress as a critical factor in nonalcoholic fatty liver disease pathogenesis. Antioxidants & RedoxSignaling, 26(10), 519–541. 10.1089/ars.2016.6776 27452109

[fsn31118-bib-0042] Stams, A. J. , & Plugge, C. M. (2009). Electron transfer in syntrophic communities of anaerobic bacteria and archaea. Nature Review Microbiology, 7(8), 568–577. 10.1038/nrmicro2166 19609258

[fsn31118-bib-0043] Topping, D. L. , & Clifton, P. M. (2001). Short‐chain fatty acids and human colonic function: Roles of resistant starch and nonstarch polysaccharides. Physiological Review, 81(3), 1031–1064. 10.1152/physrev.2001.81.3.1031 11427691

[fsn31118-bib-0044] vanWinsen, R. L. , Keuzenkamp, D. , Urlings, B. A. , Lipman, L. J. , Snijders, J. A. , Verheijden, J. H. , … van Knapen, F. (2002). Effect of fermented feed on shedding of Enterobacteriaceae by fattening pigs. Veterinary Microbiology, 87(3), 267–276. 10.1016/S0378-1135(02)00066-4 12052336

[fsn31118-bib-0045] Videla, L. A. , Rodrigo, R. , Orellana, M. , Fernandez, V. , Tapia, G. , Quiñones, L. , … Poniachik, J. (2004). Oxidative stress‐related parameters in the liver of non‐alcoholic fatty liver disease patients. Clinical Science, 106(3), 261–268. 10.1042/CS20030285 14556645

[fsn31118-bib-0046] Vrieze, A. , Van Nood, E. , Holleman, F. , Salojärvi, J. , Kootte, R. S. , Bartelsman, J. F. W. M. , … Nieuwdorp, M. (2012). Transfer of intestinal microbiota from lean donors increases insulin sensitivity in individuals with metabolic syndrome. Gastroenterology, 143(4), 913–916.e7. 10.1053/j.gastro.2012.06.031 22728514

[fsn31118-bib-0047] Wang, Z. , Xiao, G. , Yao, Y. , Guo, S. , Lu, K. , & Sheng, Z. (2006). The role of bifidobacteria in gut barrier function after thermal injury in rats. Journal of Trauma, 61(3), 650–657. 10.1097/01.ta.0000196574.70614.27 16967002

[fsn31118-bib-0048] Woodhouse, C. A. , Patel, V. C. , Singanayagam, A. , & Shawcross, D. L. (2018). Review article: The gut microbiome as a therapeutic target in the pathogenesis and treatment of chronic liver disease. Alimentary Pharmacology Therapeutics, 47(2), 192–202. 10.1111/apt.14397 29083037

[fsn31118-bib-0049] Wu, P.‐J. , Chen, J.‐B. , Lee, W.‐C. , Ng, H.‐Y. , Lien, S.‐C. , Tsai, P.‐Y. , … Chiou, T.‐Y. (2018). Oxidative stress and nonalcoholic fatty liver disease in hemodialysis patients. Biomed Research International, 2018, 1–7. 10.1155/2018/3961748 PMC6236669 30515395

[fsn31118-bib-0051] Ye, H. Q. , Li, Q. , Zhang, Z. Z. , Sun, M. C. , Zhao, C. H. , & Zhang, T. H. (2017). Effect of a novel potential probiotic *Lactobacillus paracasei* Jlus66 isolated from fermented milk on nonalcoholic fatty liver in rats. Food & Function, 8(12), 4539–4546. 10.1039/c7fo01108c 29106426

[fsn31118-bib-0052] Zhu, L. , Baker, S. S. , Gill, C. , Liu, W. , Alkhouri, R. , Baker, R. D. , & Gill, S. R. (2013). Characterization of gut microbiomes in nonalcoholic steatohepatitis (NASH) patients: A connection between endogenous alcohol and NASH. Hepatology, 57(2), 601–609. 10.1002/hep.26093 23055155

